# Study on Performance Damage and Mechanism Analysis of Asphalt under Action of Chloride Salt Erosion

**DOI:** 10.3390/ma14113089

**Published:** 2021-06-04

**Authors:** Peilei Zhou, Wensheng Wang, Lili Zhu, Haoyun Wang, Yongming Ai

**Affiliations:** 1 College of Transportation, Jilin University, Changchun 130025, China; zhoupeilei@jlu.edu.cn (P.Z.); wanghy1717@mails.jlu.edu.cn (H.W.); 2 College of Construction Engineering, Jilin University, Changchun 130025, China; 3 Capital Construction Department, Guangxi Normal University, Guilin 541004, China; zhullgnu@163.com

**Keywords:** asphalt, salt erosion, temperature sensitivity, Fourier transform infrared spectroscopy

## Abstract

This study aims to investigate the performance evolution and mechanism of asphalt under action of chloride salt erosion. Asphalt samples soaked with five different snow melting chloride salt concentrations were taken as the research object. Then, the high-temperature performance, low-temperature performance, temperature sensitivity and asphalt–aggregate adhesion property of asphalt samples were carried out. Additionally, Fourier transform infrared spectroscopy (FTIR) was used to explore the mechanism of chloride salt erosion on asphalt. Test results showed the linear variation relationships of high-temperature performance, low-temperature performance and temperature sensitivity with chloride salt concentrations. The high-temperature performance of asphalt would be improved by chloride snowmelt salt. With the increase in the chloride salt solution concentration, the low-temperature performance of asphalt became worse, and the temperature sensitivity increased. Moreover, after the effect of the chloride salt solution, the asphalt–aggregate adhesion property decreased with the increase in the chloride salt solution concentration. It is necessary to control the amount of chloride snowmelt salt in the actual snow removal projects. Finally, based on Fourier transform infrared spectroscopy, the mechanism of chloride salt erosion on asphalt was preliminarily explored. With the increase in the chloride salt solution concentration, the proportion of light components (saturated fraction, aromatic fraction) in asphalt decreased, and the proportion of heavy components (resin and asphaltene) with good thermal stability increased.

## 1. Introduction

Asphalt pavement has good skid resistance, low noise and excellent friction resistance, which has been applied as the main structure of expressway pavement in China [1,2,3]. However, asphalt pavement is prone to high temperature rutting, low temperature cracking, water damage and spalling [4,5,6,7,8,9,10]. In some special natural environments, especially in coastal areas and northwest saline soil areas, corrosion medium such as sulfate–chloride would lead to the serious damage of asphalt pavement after several years of service, and the service life of the road is greatly reduced [11,12,13,14,15]. In addition, the road surface is easy to freeze in winter. In order to keep the road smooth and retain driving safety, salt particles are sprayed directly to melt ice and snow [16,17]. In recent years, the self-snow-melting pavement, which has achieved certain development, can directly solve the problem by adding snow melting and ice suppression materials [18,19]. No matter what way, the existence of salt will bring continuous negative effects to asphalt pavement.

The method of spreading deicing salt, a snow melting agent with the function of quick deicing and snow melting, could be considered an effective method to solve the problem of traffic jams and prevent the pavement from continuing to freeze [16,20]. The theory principle of spreading deicing salt, a snow melting agent, is generally based on the freezing point reduction theory [13,15]. The so-called freezing point refers to the temperature when a substance reaches the state of solid–liquid coexistence and two-phase equilibrium. When chloride salt ions are added into water, the surface of the water is occupied by chloride salt ions, resulting in a relatively small vapor pressure of water compared with ice. Then, it is necessary to lower the temperature to make the vapor pressure of ice equal to the vapor pressure of ice, resulting in a decrease in the freezing point. Firstly, many scholars have explored the macro performance changes of asphalt materials in the salt solution environment. Xiong et al. carried out the durability evaluation of an asphalt mixture in the salt corrosion condition under the effect of dynamic water pressure by the splitting test [12]. Wang et al. investigated the pavement performances of an asphalt mixture containing salt-storage aggregates with the function of snow melting [16]. Amini et al. investigated the influences of moisture and chloride salt on the performance degradation of asphalt mixture subjected to freeze–thaw cycles according to the Marshall stability and mass loss [21]. Feng et al. discussed the effects of salt on an asphalt binder based on the conventional performances, and further studied the influences of salt and freeze–thaw cycles on the mechanical and volume properties of an asphalt mixture [22]. The above research shows that the freeze–thaw cycle effect is the main influence factor of asphalt mixture damage, and chloride salt erosion would accelerate the damage of asphalt mixtures.

The influence of salt erosion on asphalt material is not only reflected in the macro performance of asphalt mixture, but also reflected in the performance change of asphalt itself. Zhang et al. studied the influences of salt erosion conditions on the asphalt binder by using a four fractions test and atomic force microscopy, and they found that the main reason for the performance degradation of an asphalt binder is that the chemical composition of the asphalt binder changed under the action of salt erosion, resulting in the phenomenon of salt aging. At the same time, the freeze–thaw cycle test of a salt solution was simulated [23]. Zhang et al. studied the influences of chlorine salt and freeze–thaw cycles on asphalt mastics from the perspective of microscopic characteristics by using Fourier transform infrared spectroscopy, gel permeation chromatography, and atomic force microscopy techniques [24]. The above research shows that salt erosion can significantly reduce the performance of asphalt itself, leading to the decrease in the bonding effect of the asphalt binder, and then affect the adhesion between the asphalt and aggregate. Under the influence of long-term salt erosion and other external environmental factors, the strength of the asphalt material will reduce rapidly.

Considering the problem that the existence of salt would bring continuous negative effects to asphalt pavement, this study aims at analyzing the performance evolution and mechanism of asphalt under action of chloride salt erosion. Asphalt samples soaked with different snow melting chloride salt concentrations are taken as the research object. The softening point and viscosity are used to evaluate the high-temperature performance of asphalt, the ductility and equivalent brittle point are used to evaluate the low-temperature performance, and the penetration index (*PI*) is used to evaluate the temperature sensitivity of asphalt. In addition, the asphalt–aggregate adhesion property is evaluated, and the mechanism of chloride salt erosion on asphalt is preliminarily explored by Fourier transform infrared spectroscopy (FTIR). The analysis and results of this study could provide some references for the snow melting project by spreading deicing salt, a snow melting agent.

## 2. Materials and Methods

### 2.1. Experimental Materials

#### 2.1.1. Asphalt

In this study, the base asphalt was asphalt with a penetration of 70 (×0.1 mm) (Zhonghai Asphalt Co., Ltd., Binzhou, China), which meets the requirements of Chinese specification “Technical specifications for construction of highway asphalt pavements” (JTG F40-2004) [25]. The detailed performance indexes are shown in Table 1.

#### 2.1.2. Snowmelt Salt

**Figure 1:**
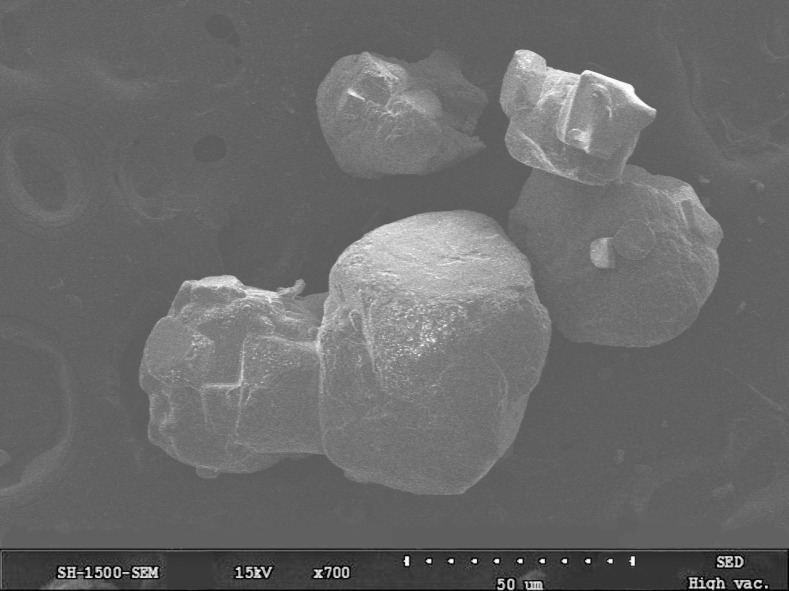
The SEM image of snow melting chloride salt.

**Figure 2:**
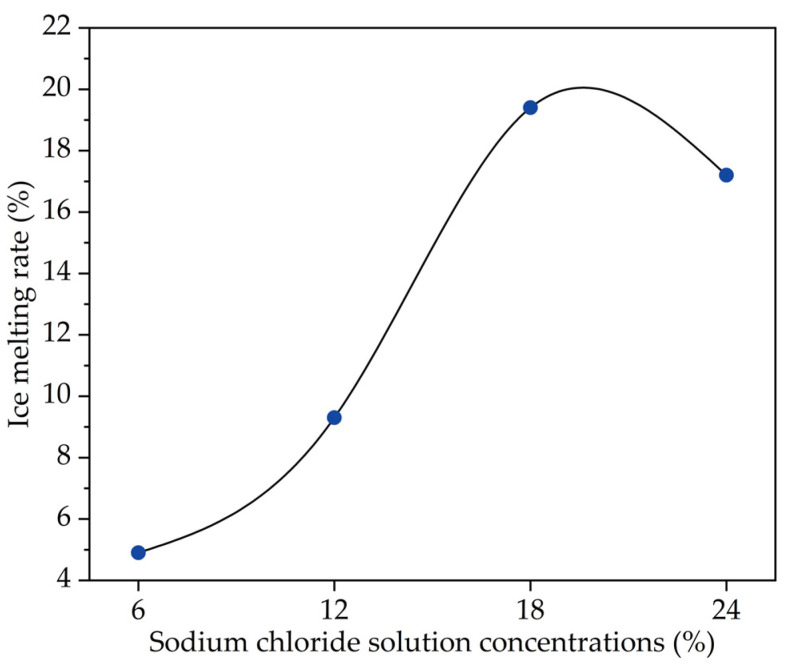
Snow melting ability of chloride salt solutions with concentrations.

This study used sodium chloride snow melting salt to investigate the influence of chloride salt concentration on the performance of asphalt. Following the Chinese specification of “Salt of ice and snow melting for road” (GB/T 23851-2009) [26], the dissolution rate of chloride salt (snow melting salt) is 6.955 g/min, which meets the specification requirements. In general, the concentration of snow melting chloride salt determines the deicing performance to a large extent. The SEM image of snow melting chloride salt is shown in Figure 1. Figure 1 shows that the surface of snow melting chloride salt is relatively smooth with few particles attached, and it is easy to connect with water molecules [27]. At the same time, snow melting chloride salt would be easier to enter into the interior of pavement materials [27]. In order to explore the deicing ability of snow melting chlorine salt at different concentrations, the following experiment was designed for analysis: (1) an amount of 100 mL of water was added into a container of the same size and placed at −10 °C in a low temperature incubator until freezing. (2) After 12 h, different chloride salt solutions with concentrations of 6%, 12%, 18% and 24% were poured into four containers with ice cakes. (3) These containers with ice cakes and chloride salt solutions were placed in constant temperature environment at 0 °C for 0.5 h, and the ice melting volume could be measured. The ice melting rate is calculated by the ratio of ice melting volume to ice volume. The snow melting ability of chloride salt solutions with different concentrations of is shown in Figure 2.

It can be seen from Figure 2 that with the increase in the chloride salt solution concentration, the ice melting rate also increases, implying the ice melting ability of chloride solution gradually increases. When the chloride salt solution concentration reaches a certain value, the ice melting ability also reaches the peak, and then the ice melting rate decreases with the increase in chloride solution concentration. This is because the chloride salt solution reaches a saturation state, the chloride salt cannot continue to dissolve when the chloride concentration continues to increase, and the chloride solute precipitates gradually, which affects the ice melting rate. Therefore, it is very important to study the influence of different chloride salt solution concentrations on the performances of asphalt, which would be essential to reduce the damage of snowmelt salt on asphalt pavement.

#### 2.1.3. Asphalt Sample Preparation

The interaction between asphalt and chloride salt solution is a slow and long process. In order to speed up the interaction rate of these two substances, increasing temperature is usually used to accelerate their interaction. In this study, the high temperature boiling method was used to prepare asphalt samples following the previous study [28]. The specific steps are as follows:

- Step 1:

Chloride salt solutions with different concentrations (0%, 6%, 12%, 18% and 24%) were poured into the crucible for heating and boiling, then the heated asphalt with a molten state were poured into the chloride salt solutions and boiled for 3 min.

- Step 2:

The surface of the prepared porcelain basin was evenly smeared with isolation agent, and the boiled asphalt was taken out and placed in the porcelain basin and marked.

- Step 3:

The porcelain basin containing asphalt was placed on the electric stove at 120~130 °C to separate moisture from the asphalt until the asphalt is not foamed.

### 2.2. Experimental Methods

#### 2.2.1. High-Temperature Performance by Softening Point and Viscosity Tests

The ability of asphalt to resist high temperature deformation is usually known as high-temperature stability. In the current test specification “Standard test methods of bitumen and bituminous mixtures for highway engineering” (JTG E20-2011) [29], the ball and ring method (T0606-2011) [29] is used to determine the softening point of asphalt. The higher the softening point of asphalt, the better the high-temperature performance of asphalt. On the other hand, because of the wide temperature range of asphalt, the viscosity range of asphalt is also very large. When heating and melting, the viscosity of asphalt may be as low as 10^−1^ Pa·s, while the viscosity of asphalt would be as high as 10^11^ Pa·s in the severe cold condition. In general, the road surface temperature is very high in summer in China, so the viscosity of asphalt at 135 °C (T0625-2011) [29] is regarded to truly reflect the performance of asphalt. Therefore, these two commonly used indexes, i.e., softening point and viscosity, were used to evaluate the high-temperature performance of asphalt [30].

#### 2.2.2. Low-Temperature Performance by Ductility and Equivalent Brittle Point Tests

The crack problem of asphalt pavement has attracted extensive attention; the main reason for this problem is the lack of low-temperature crack resistance of the asphalt mixture [31]. Asphalt plays a bonding role in the asphalt mixture, and its low-temperature performance has an important impact on the low-temperature crack resistance of asphalt pavement. Then, it is suggested that the ductility at 10 °C (T0605-2011) [29] and the equivalent brittle point T_1.2_ (T0604-2011) [29] can be used as the evaluation indexes of the low-temperature performance of asphalt in this study.

#### 2.2.3. Temperature Sensitivity by Penetration Index

As a kind of typical temperature-sensitive material, the temperature change has a significant impact on the performance of asphalt. As for the index of temperature sensitivity of asphalt, the most representative parameters are penetration–viscosity index (PVN), viscosity–temperature index (VTS) and *PI* [30]. In this study, the *PI* of asphalt is used to evaluate the temperature sensitivity of asphalt. The *PI* value could be calculated by three or more penetration values of asphalt tested under different temperature conditions according to the specified method (T0604-2011) of “Standard test methods of bitumen and bituminous mixtures for highway engineering” (JTG E20-2011) [29]. The greater the *PI* value, the smaller the temperature sensitivity of asphalt.

#### 2.2.4. Asphalt–Aggregate Adhesion Property Test

Water damage is the main early damage form of asphalt pavement among the common distresses in China. The existence of water will destroy the bonding interface between the asphalt and aggregate, which leads to the asphalt film falling off from the aggregate surface, and then the road performance of asphalt pavement will be decreased [32]. At present, the boiling method and water immersion method are the most widely used tests to evaluate the adhesion property between the asphalt and aggregate in China [33]. In this study, according to T0616-2011 of “Standard test methods of bitumen and bituminous mixtures for highway engineering” (JTG E20-2011) [29], the boiling method was used to test the effect of the chloride salt solution concentration on the asphalt–aggregate adhesion property between asphalt and aggregate.

#### 2.2.5. Fourier Transform Infrared Spectroscopy Test

Infrared spectroscopy (IR) can react with the molecular structure of materials, which can effectively detect the structure of complex molecules [34,35]. In this study, according to “General rules for infrared analysis” (GB/T 6040-2002) [36], the infrared spectroscopy of asphalt samples soaked in different chloride salt solution concentrations were measured by Fourier transform infrared spectrometer to observe the changes of the molecular structure of asphalt.

## 3. Results and Discussion

### 3.1. High-Temperature Performance

#### 3.1.1. Softening Point

**Figure 3:**
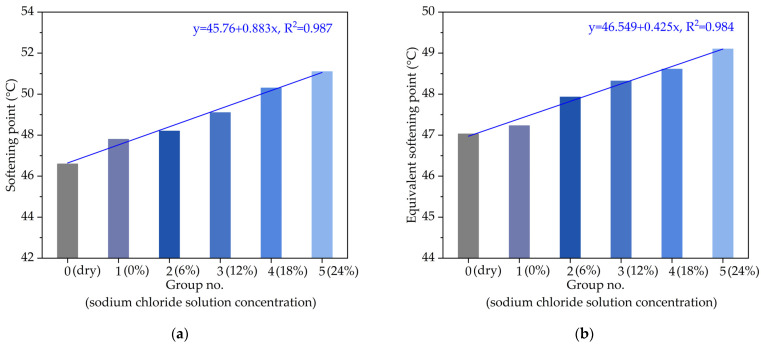
The variation of high-temperature performance with different chloride salt concentrations: (**a**) softening point; (**b**) equivalent softening point.

According to the ball and ring method (T0606-2011) of Chinese specification (JTG E20-2011), the measured softening point results of asphalt with different chloride salt concentrations are plotted in Figure 3a. When the penetration of asphalt is 800 (0.1 mm), the corresponding temperature is known as the equivalent softening point T_800_, which can also be used to evaluate the high-temperature performance of asphalt. The equivalent softening point T_800_ can be calculated by extending the fitting linear penetration–temperature line at the intersection of penetration value 800 in the nomograph with three or more penetration values of asphalt under different temperature conditions. The calculation equation is listed in the Equation (1). The calculated equivalent softening point T_800_ of asphalt with different chloride salt concentrations are plotted in Figure 3b.

(1) $$T_{800}=\frac{2.9031-K}{A_{\mathrm{lg}P}}$$

in which *K* is the fitted constant in the regression equation of penetration–temperature, and *A*_lg*P*_ is the fitted coefficient in the regression equation of penetration–temperature. The fitting results are listed in Table 2.

It can be seen from Figure 3 that from the overall trend, the softening point and equivalent softening point T_800_ of asphalt samples soaked in the chloride salt solution are significantly higher than those of the dry asphalt sample. In Figure 3a, the softening point of the asphalt sample soaked in water (Group No. 1) is 2.5% higher than that of the asphalt sample in Group No. 0. Besides, when the chloride salt solution concentration increases from 6% to 24%, the softening point of asphalt samples increases by 3.5%, 5.4%, 8.0% and 9.6%, respectively. In Figure 3b, compared with the control group (Group No. 0), the equivalent softening point T_800_ of the asphalt sample after soaking in water and chloride salt solution with various concentrations increased by 0.4%, 1.8%, 2.6%, 3.7% and 4.3%, respectively. Both the softening point and equivalent softening point show linear relationships with the increase in chloride salt concentration. The variation of the softening point and equivalent softening point with different chloride salt concentrations shows that under the effect of chloride snowmelt salt, the softening point of asphalt would increase, and the greater the chloride salt solution concentration is, the larger the change range of the softening point is, which indicated that the high-temperature performance of asphalt would be improved by chloride snowmelt salt.

For these reasons, on the one hand, due to the change of asphalt components, with the increase in the chloride salt solution concentration, the proportion of light components (saturated fraction, aromatic fraction) in asphalt decreases, and the proportion of heavy components (resin and asphaltene) with good thermal stability increases, which would lead to the increase in high-temperature performance of asphalt. On the other hand, the chloride ions in the chloride salt solution exist in the form of crystals among asphalt molecules and increase the molecular weight of asphalt. At this time, asphalt molecules need to overcome more resistance while moving. In addition to the heat absorbed by asphalt molecules, chloride ions would share part of the heat. Then, with the increase in the chloride salt solution concentration (i.e., the chloride ions increase), the asphalt heating system including the chloride salt solution could bear more heat, resulting in a better high-temperature performance of asphalt.

#### 3.1.2. Viscosity

**Figure 4:**
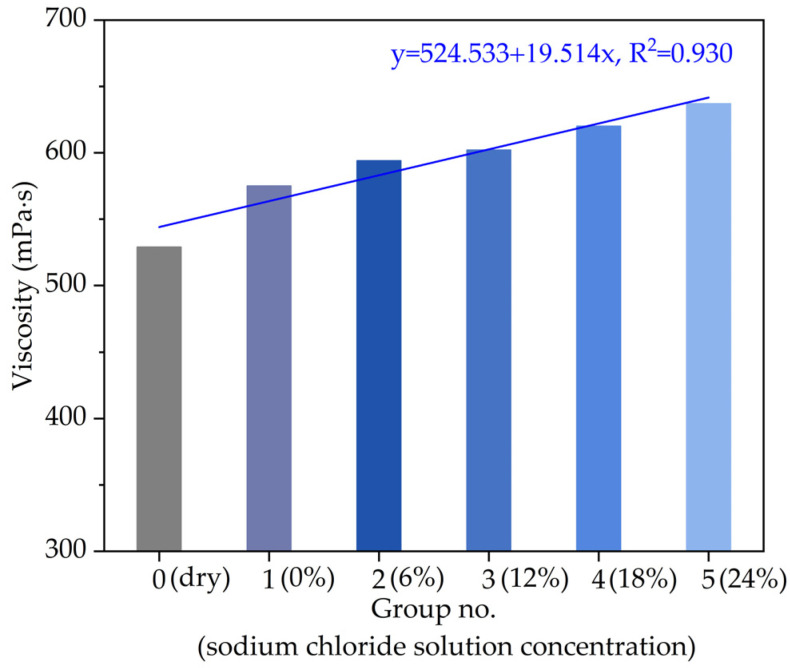
The viscosity variation of asphalt at 135 °C with different chloride salt concentrations.

In order to further analyze the influence of chloride snowmelt salt on the high-temperature performance of asphalt, in addition to the softening point, the viscosity of asphalt at 135 °C were also measured according to T0625-2011 of Chinese specification (JTG E20-2011). The viscosity variation of asphalt at 135 °C with different chloride salt concentrations is shown in Figure 4.

As can be seen from Figure 4, compared with the control group (Group No. 0), the viscosity at 135 °C of asphalt soaked in water and chloride salt solutions is improved on the whole. The viscosity variation of asphalt at 135 °C varying with chloride salt solution concentrations presents a linear trend with R^2^ of 0.930. When the chloride salt solution concentration increases from 0% to 24%, compared with the control group (Group No. 0), the viscosity at 135 °C of asphalt samples increase by 5.2%, 7.9%, 12.1%, 14.1% and 17.9%, respectively. The viscosity variation trend also indicates that the high-temperature performance of asphalt sample has been improved by chloride snowmelt salt. Due to the existence of chloride ions in asphalt molecules, in the Brookfield viscometer test, the change of asphalt composition (the increase in resin and asphaltene contents with larger molecular weight) requires the rotor to overcome a greater intermolecular force in order to maintain a fixed speed. Therefore, there is an increasing viscosity value of asphalt, and the viscosity increasement in asphalt will increase with the increase in the chloride solution concentration.

### 3.2. Low-Temperature Performance

#### 3.2.1. Ductility

**Figure 5:**
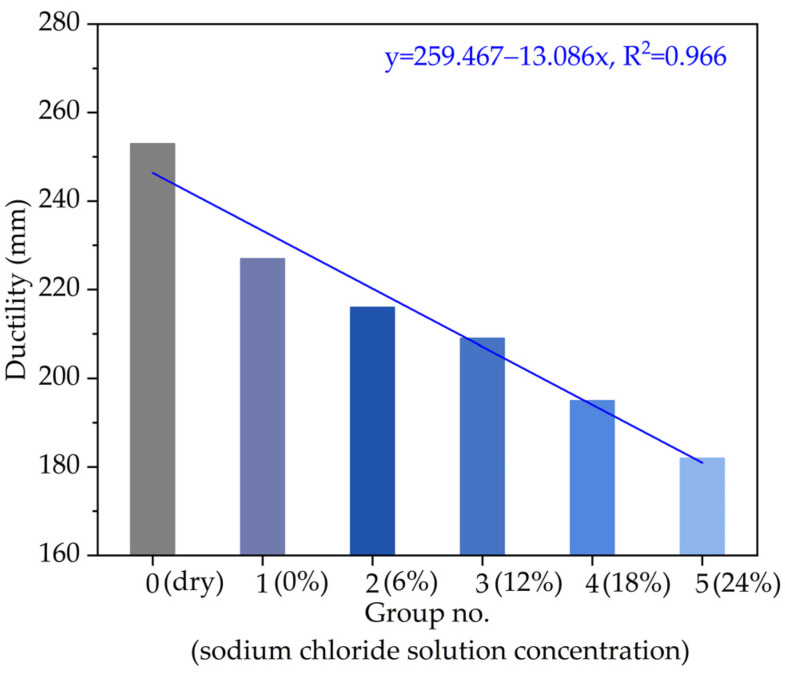
The variation of ductility at 10 °C with different chloride salt concentrations.

According to the experimental method (T0605-2011) of Chinese specification (JTG E20-2011), the measured ductility results at 10 °C of asphalt with different chloride salt solution concentrations are plotted in Figure 5. As can be seen from Figure 5, with the increase in the chloride salt solution concentration, the ductility at 10 °C of the asphalt sample presents a strong linear variation trend with R^2^ of 0.966. Compared with the control group (Group No. 0), the ductility at 10 °C of the asphalt sample decreases by 9.2%, 13.6%, 17.2%, 23.3% and 27.6% when the chloride salt solution concentration increases from 0% to 24%, respectively. The ductility variation of asphalt depends on the chloride salt solution concentration. The ductility at 10 °C of the asphalt soaked in a higher concentration of chloride salt solution is significantly lower than that of the control group (dry asphalt sample).

The main reason for such ductility variation of asphalt with different chloride salt solution concentrations is that the chloride ions in the chloride salt solution process crystals with large sizes among asphalt molecules, leading to materials being easier to break, and the chloride salt ions will block the links between asphalt molecules, which makes asphalt easier to break in the ductility test. With the increase in the chloride salt solution concentration (i.e., the chloride ions increase), the low-temperature performance of asphalt becomes worse. In addition, with the increase in the chloride salt solution concentration, the asphalt components gradually change, the proportion of heavy components (resin and asphaltene) increases, while the proportion of light components (saturated fraction, aromatic fraction) with good plasticity in asphalt decreases, which would lead to a worse ductility of asphalt. Moreover, the ductility at 10 °C of the asphalt is generally required no less than 200 mm. According to the ductility result in Figure 5, the chloride salt solution concentration should be recommended as less than 12~18%.

#### 3.2.2. Equivalent Brittle Point

When the penetration of asphalt is 1.2 (0.1 mm), the corresponding temperature is known as the equivalent brittle point T_1.2_, which can also be used to evaluate the low-temperature performance of asphalt. The equivalent brittle point T_1.2_ is also calculated by extending the fitting linear penetration–temperature line at the intersection of penetration value 1.2 in the nomograph with three or more penetration values of asphalt under different temperature conditions. The corresponding calculation equation is listed in the Equation (2).

(2) $$T_{1.2}=\frac{0.0792-K}{A_{\mathrm{lg}P}}$$

**Figure 6:**
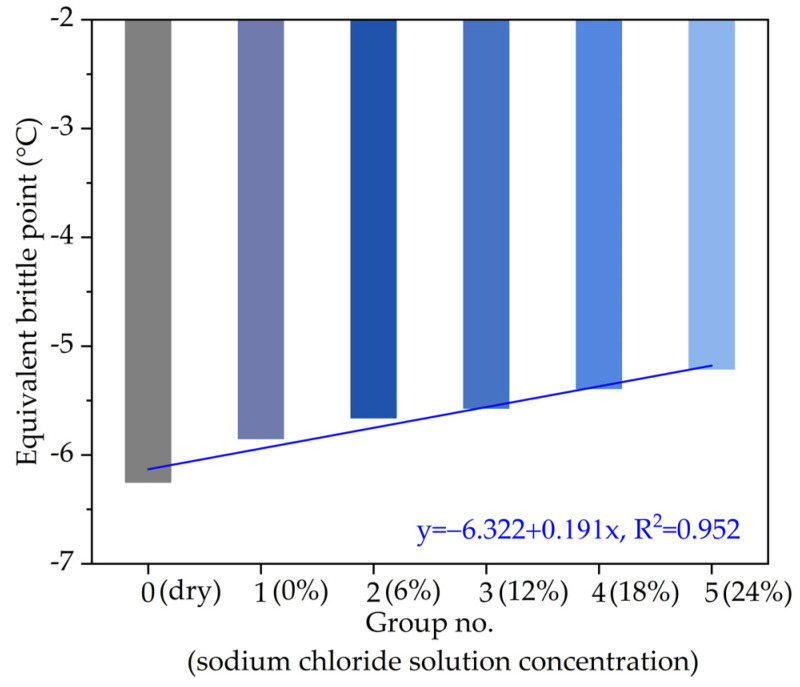
The variation of equivalent brittle point with different chloride salt concentrations.

According to the fitting parameters in Table 2, the calculated equivalent brittle points T_1.2_ of asphalt with different chloride salt concentrations are plotted in Figure 6. From the overall variation trend in Figure 6, the equivalent brittle point T_1.2_ of asphalt sample presents a linear relationship with the increase in the chloride salt solution concentration, in which R^2^ of 0.966. Similarly, the chloride ions in the chloride salt solution contain crystals with large sizes among asphalt molecules, which have poor plasticity. Due to the change of asphalt components, the equivalent brittle point T_1.2_ of asphalt decreases with the chloride salt solution concentration.

### 3.3. Temperature Sensitivity

According to the experimental method (T0604-2011) of Chinese specification (JTG E20-2011), the penetration values at 15 °C, 25 °C and 30 °C of asphalt could be measured. Then, the measured penetration–temperature result is fitted by a linear function. Following the T0604 in JTG E20-2011, the calculation equation of *PI* is listed in the Equation (3), which is used to evaluate the temperature sensitivity of asphalt.

(3) $$PI=\frac{20-500A_{\mathrm{lg}P}}{1+50A_{\mathrm{lg}P}}$$

**Figure 7:**
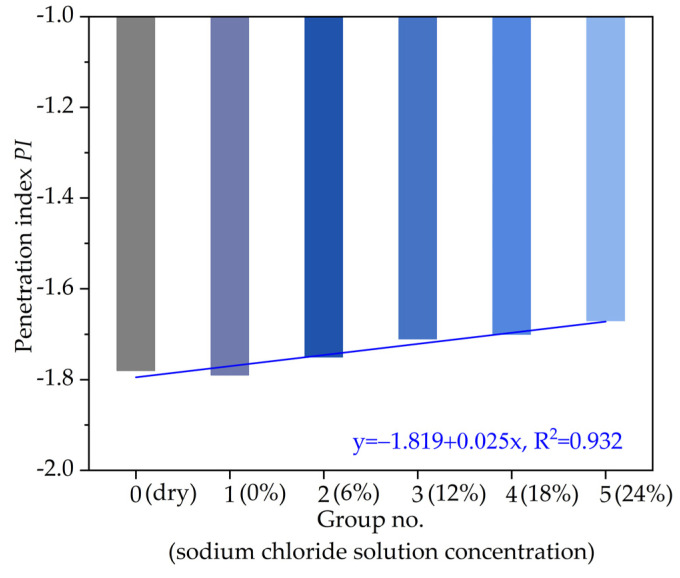
The variation of *PI* with different chloride salt concentrations.

Following the fitting parameters in Table 2, the calculated *PI* of asphalt with different chloride salt concentrations are plotted in Figure 7. In Figure 7, from the overall variation trend, the temperature sensitivity of asphalt decreases slightly by water, and the temperature sensitivity increases by soaking in chloride salt solution concentration. The temperature sensitivity of asphalt sample presents an increasing linear relationship with the increase in the chloride salt solution concentration and R^2^ of 0.932, which indicates that the higher the chloride salt solution concentration, the greater the *PI* value of asphalt, and the better the temperature sensitivity. The reason is that: after the action of chloride salt solution, the composition of asphalt changes; that is, the proportion of light components (saturated fraction, aromatic fraction) in asphalt decreases, and the proportion of heavy components (resin and asphaltene) with good thermal stability increases. Besides, with the increase in the chloride salt solution concentration, the content of resin and asphaltene in asphalt samples will be further increased, so the temperature sensitivity of asphalt will be reduced. Moreover, the chloride ions in the chloride salt solution exist in the form of crystals among asphalt molecules; when the asphalt heating system including chloride salt solution absorb heat to move, the chloride ions would share part of the heat.

### 3.4. Asphalt–Aggregate Adhesion Property

**Figure 8:**
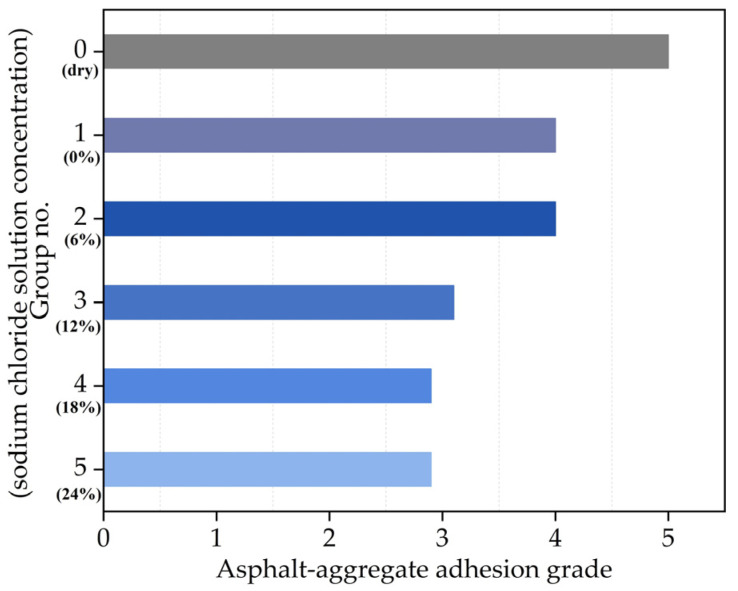
The asphalt–aggregate adhesion grade of asphalt with different chloride salt concentrations.

The boiling method was adopted in this study to evaluate the adhesion property between asphalt and aggregate according to T0616-2011 of JTG E20-2011 (similar to ASTM D3625). The limestone aggregates were used in this study, the corresponding geological nature and some characteristics have been introduced in the previous study [28]. The asphalt–aggregate adhesion grade of asphalt varying with different chloride salt concentrations is shown in Figure 8.

It can be seen in Figure 8 that the adhesion between asphalt and aggregate decreases after the effect of the chloride salt solution, and the spalling area of asphalt film generally increases with the increase in the chloride salt solution concentration. In other words, the asphalt–aggregate adhesion property decreases with the increase in the chloride salt solution concentration. The reason for the above variation result is that after the asphalt is soaked in chloride salt solution, the moisture penetrates into asphalt through adsorption, replacement and diffusion, causing damage to the bonding interface between asphalt and aggregate. In addition, the sodium ions in chloride salt solution have chemical adsorption with asphalt, which accelerates asphalt emulsification and makes asphalt unable to adhere to the aggregate surface. With the increase in the chloride salt solution concentration, more and more sodium ions have adsorption with asphalt, resulting in the poor asphalt–aggregate adhesion. There are many rainy and snowy days in the north of China in winter. Generally, the requirement for the asphalt–aggregate adhesion grade of expressway and first-class highway is grade 4. It can be seen from Figure 8 that if the chloride salt solution concentration is higher than 12%, asphalt pavement finds it difficult to meet the requirement of the asphalt-adhesion grade. Therefore, it is necessary to control the amount of chloride snowmelt salt in the actual snow removal projects.

### 3.5. Fourier Transform Infrared Spectroscopy Test

**Figure 9:**
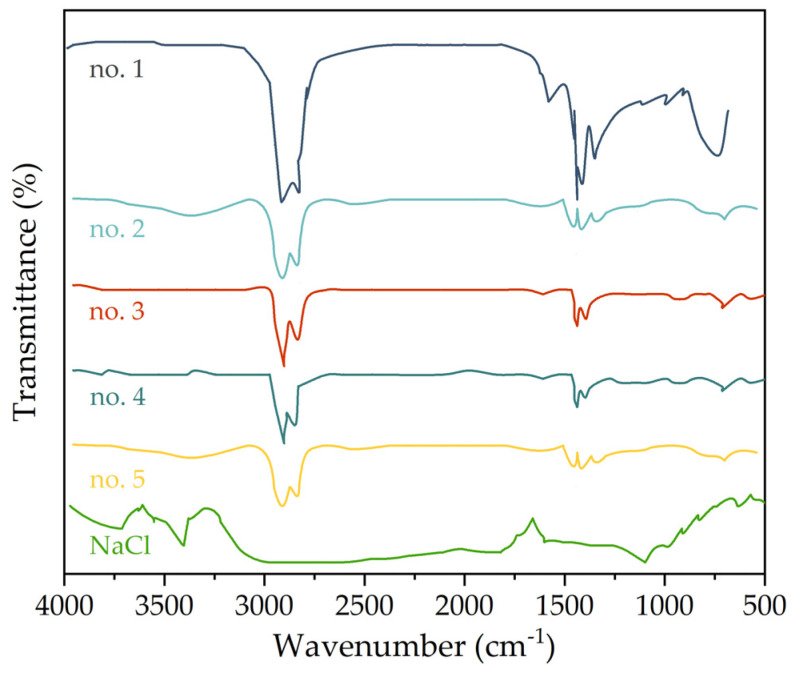
The FTIR results of asphalt with different chloride salt concentrations.

Fourier transform infrared spectroscopy (FTIR) was used to test asphalt samples soaked in different chloride salt solution concentrations to analyze the action mechanism of chloride salt on asphalt. The FTIR results of asphalt varying with different chloride salt concentrations is shown in Figure 9. As can be seen in Figure 9, compared with the base asphalt soaked in water, the absorption peak in the characteristic area of asphalt samples soaked in chloride salt solution has no change. The main absorption peaks of asphalt samples in different chloride salt solution concentrations are as follows: the antisymmetric stretching vibration of methylene (CH_2_) at 2918 cm^−1^, symmetric stretching vibration of methylene (CH_2_) at 2850 cm^−1^, skeleton vibration of benzene ring near 1460 cm^−1^, shear vibration of methyl (CH_3_) near 1378 cm^−1^, and benzene ring substitution area of in the wavenumber range of 900~500 cm^−1^. The addition of chloride salt has a great influence on the absorption peak near 1600 cm^−1^ characterizing aromatic fraction and the benzene ring substitution area, which would be also the main reason for the performance change of asphalt caused by chloride salt addition.

## 4. Conclusions

This study takes the asphalt samples soaked with different snow melting chloride salt concentrations as the research object. The softening point and viscosity are used to evaluate the high-temperature performance of asphalt, the ductility and equivalent brittle point are used to evaluate the low-temperature performance, and the *PI* is used to evaluate the temperature sensitivity of asphalt. In addition, the asphalt–aggregate adhesion property is evaluated, and the mechanism of chloride salt erosion on asphalt is preliminarily explored by Fourier transform infrared spectroscopy (FTIR). The conclusions are as follows:

1. The linear variation relationships of the softening point, equivalent softening point and viscosity with chloride salt concentrations showed that the high-temperature performance of asphalt would be improved by chloride snowmelt salt.
2. According to the ductility and equivalent brittle point, with the increase in the chloride salt solution concentration (i.e., the chloride ions increase), the low-temperature performance of asphalt becomes worse, and the chloride salt solution concentration should be recommended as less than 12~18%.
3. The temperature sensitivity of asphalt presents an increasing linear relationship with the increase in the chloride salt solution concentration, and the temperature sensitivity increases by soaking in chloride salt solution concentration.
4. After the effect of the chloride salt solution, the asphalt–aggregate adhesion property decreases with the increase in the chloride salt solution concentration. It is necessary to control the amount of chloride snowmelt salt in the actual snow removal projects.
5. Based on Fourier transform infrared spectroscopy, the main reason for the performances of asphalt caused by chloride salt might be attributed to the change of asphalt components. With the increase in the chloride salt solution concentration, the proportion of light components (saturated fraction, aromatic fraction) in asphalt decreases, and the proportion of heavy components (resin and asphaltene) with good thermal stability increases.

In future work, the influence of this salt contents variations along time (including aging simulation, alternated with UV radiation) would be evaluated. Meanwhile, further study will carry out the characterization tests (such as SEM or TEM) for the used materials, and the rheological property test of asphalt.

## Figures and Tables

**Table 1:** Basic performance parameters of AH-70 asphalt.

Test Items	Units	Index	Standards	Requirements
Penetration @ 25 °C	0.1 mm	76	T0604	60~80
Penetration index (*PI*)	-	−1.78	T0604	−1.8~+1.0
Ductility @ 10 °C	cm	25.3	T0605	≥20
Ductility @ 15 °C	cm	>100	T0605	≥100
Softening point (R and B)	°C	46.6	T0606	≥43
Brookfield viscosity @ 135 °C	Pa∙s	0.529	T0625	-
Brookfield viscosity @ 60 °C	Pa∙s	0.794	T0625	-

Note: Standards and requirements are defined in Chinese technical specifications (JTG F40-2004).

**Table 2:** Fitting parameters of penetration–temperature linear relationship.

Group No.	0 (Dry)	1 (0%)	2 (6%)	3 (12%)	4 (18%)	5 (24%)
*K*	0.05302	0.05318	0.05268	0.05256	0.05238	0.05211
*A*_lg*P*_	0.41102	0.39052	0.37755	0.37223	0.36230	0.34891
R^2^	0.997	0.999	0.999	0.998	0.999	0.999

## Data Availability

The data presented in this study are available on request from the corresponding author.
